# Food Addiction Beliefs Amongst the Lay Public: What Are the Consequences for Eating Behaviour?

**DOI:** 10.1007/s40429-017-0136-0

**Published:** 2017-05-10

**Authors:** Helen K. Ruddock, Charlotte A. Hardman

**Affiliations:** Department of Psychological Sciences, University of Liverpool, Liverpool, UK

**Keywords:** Food addiction, Perceptions and beliefs, Food reward, Eating behaviour

## Abstract

**Purpose of Review:**

The current paper reviews recent research on perceptions of food addiction in the lay public. It also examines the potential consequences of such beliefs for eating behaviour.

**Recent Findings:**

Surveys suggest that, within community samples, the majority of individuals believe that certain foods are addictive, and that food addiction causes obesity. Further, many people believe *themselves* to be ‘food addicts’, and these individuals demonstrate increased patterns of aberrant eating. However, there is also initial experimental evidence to suggest that believing oneself to be a food addict leads to short-term food restriction.

**Summary:**

To reconcile these findings, a self-perpetuating relationship between food addiction beliefs and aberrant eating is proposed. Specifically, in the short term, food addiction beliefs may encourage individuals to avoid certain foods. However, attempts at restriction may eventually lead to increased cravings and disinhibition, thus reinforcing perceptions of oneself as a food addict. These possibilities merit scrutiny in future research.

## Introduction

Scientific interest in the potential ‘addictive’ properties of certain foods dates back to the late nineteenth century [1•]. In 1956, Theron Randolph introduced the term ‘food addiction’ suggesting that certain foods produce a “common pattern of symptoms descriptively similar to those of other addictive processes” ([2], p. 221). Since then, much research has focused upon elucidating the biological and behavioural similarities between the effects of drugs and food (e.g. [3, 4]). In line with this, the Yale Food Addiction Scale (YFAS; 5, 6) provides a method of quantifying and ‘diagnosing’ food addiction using the clinical criterion on the DSM IV/5 for substance dependence.

However, within the scientific community, there exists substantial debate surrounding the validity of the food addiction concept. For example, Ziauddeen et al. [7] discuss the limited applicability of the DSM IV/5 substance-dependence criteria to the assessment of eating behaviour, and others point out important differences between the effects of drugs and food [8]. Nonetheless, while *scientific* opinion of the food addiction concept has been extensively reviewed, there has been relatively little consideration of the way in which food addiction is conceptualised and understood by members of the lay public. This is important because people’s beliefs about food addiction and weight gain may have far-reaching consequences on dietary behaviours, weight-related stigma, and support for obesity treatments [9•, 10]. Therefore, the focus of the current review was to provide an overview of recent research which has explored the lay public’s perceptions of food addiction and to consider whether these beliefs may have helpful or counterproductive effects on eating behaviour. Finally, we propose a self-perpetuating relationship between food addiction beliefs and longer-term patterns of dietary behaviour and provide suggestions for future research.

## The Popularity of the Food Addiction Concept

The concept of food addiction is widely endorsed amongst members of the lay public. This is reflected by the plethora of books, magazine articles, and self-help groups all dedicated to ‘curing’ people of their addiction to food. In one survey, as many as 86% of an Australian and American sample believed that certain foods are addictive, and over two thirds reported being aware of scientific evidence for comparable neurobiological effects of food and drugs [11]. Similarly, a survey of over 5000 members of a North American health organisation found that 66% believed that certain foods are addictive, and these beliefs were most prevalent amongst those with increased weight or binge eating tendencies [12]. In particular, foods that are high in fat, salt, and sugar are believed to yield the greatest addictive potential. Indeed, Lee et al. [11] found that 75% of respondents perceived sugar to be as addictive as cocaine, and foods such as chocolate, pizza, and ice cream are amongst the most commonly reported ‘problematic’ foods [13•, 14]. Furthermore, an addiction to these foods is widely believed to underlie many cases of obesity [11], and in a survey of 1000 Americans, food addiction was one of the most frequently endorsed explanations for the increasing rates of obesity in Western society [15].

## Self-Perceived Food Addiction

As well as more general support for the food addiction concept, there is evidence to suggest that many people believe *themselves* to be addicted to food. Indeed, self-perceived food addiction was reported in over half of participants from a university student sample [16] and in 27 to 42% of participants from community samples in the UK [17, 18••]. Moreover, in a survey conducted with users of a self-help website for overweight adolescents, two thirds believed themselves to be addicted to at least one food [19].

Notably, the prevalence of self-perceived food addiction amongst the general population is somewhat higher than the number of people who fulfil the YFAS diagnostic criterion for food addiction (typically 5–16%) [6, 17, 20]. In particular, in a recent study, we found that only 12% of self-perceived food addicts met this criterion [21•]. This may reflect fundamental differences between the lay public’s conceptualisation of food addiction and that proposed by clinical models of substance-dependence upon which the YFAS is based. To address this possibility, it is necessary to consider the way in which food addiction is commonly conceptualised within the lay public.

## How Is Food Addiction Defined by the Lay Public?

Amongst members of the lay public, food addiction appears to be associated with a number of core behavioural features. For example, in a sample of low-income women, Malika et al. [22] found that food addiction was characterised by a tendency to always have food available and to go out of one’s way to obtain food (e.g. shopping at odd hours in the night). Food addiction was considered distinct and more severe than food cravings; all addictive foods could be craved, but not all craved foods were thought to be addictive. Furthermore, the presence (or absence) of certain behaviours appears to be used to draw conclusions about one’s own ‘food addiction’ status. In a previous study, Hetherington and Macdiarmid [23] asked a group of self-perceived ‘chocolate addicts’ to indicate why they perceived themselves to be addicted. The majority of participants referred to an inability to resist chocolate. More specifically, they felt unable to moderate their chocolate consumption and also felt unable to stop eating chocolate once they had started.

We have recently extended these findings by providing insight into the eating-related cognitions and behaviours which are associated with a self-perceived addiction to food in general (i.e. not just chocolate) [18••]. In this study, participants from a community sample completed a brief online questionnaire in which they were asked whether or not they perceived themselves to be ‘food addicts’ and to provide the reasons behind their response. Participants’ reasons were analysed thematically, and we identified six core behaviours that were commonly attributed to an addiction to food. These included (1) reward-driven eating (i.e. eating for the rewarding aspects of food or to alleviate negative emotional states, as opposed to physiological hunger), (2) a functional or psychological preoccupation with food (i.e. a lot of time spent thinking about food, shopping, cooking, planning meals, etc.), (3) a perceived lack of self-control around food (i.e. an inability to resist tempting foods or stick to a strict diet plan), (4) frequent food cravings, (5) increased weight or an unhealthy diet, and (6) a problem with a specific type of food, particularly foods that were high in fat, salt, and sugar. Importantly, the themes identified did not differ between self-perceived food addicts and nonaddicts. Instead, self-perceived food addicts and nonaddicts reported *opposite* behaviours. For example, while self-perceived food addicts said that they “think about food all of the time”, nonaddicts reported “little interest in food”. Furthermore, the themes generated did not differ as a function of weight status or age, although females were more likely than males to attribute food addiction to a tendency to engage in reward-driven eating. Taken together, these findings suggest that food addiction is perceived to be identifiable through a core set of behaviours, and that these causal attributions are mostly consistent throughout the lay community.

These perceptions of food addiction share some notable similarities with the clinical criterion for substance dependence [5, 6]. For example, the experience of food cravings reflects a ‘persistent desire’ (in the DSM IV) and ‘craving or strong desire/urge to use a substance’ (in the DSM 5). Similarly, the tendency to consume an unhealthy diet is analogous to the DSM criterion in which ‘use continues despite knowledge of adverse consequences’. However, in contrast to the clinical criterion for substance dependence, few participants in our study referred to symptoms of tolerance (i.e. requiring increasing amounts of food over time) and withdrawal, and no participants made causal attributions to giving up ‘important social, occupational, or recreational activities’ as a result of their eating [18••]. Furthermore, participants did not associate food addiction with ‘significant distress’ or an ‘impairment to daily functioning’. These differences may underlie the aforementioned discrepancy between the widespread prevalence of self-perceived food addiction and the relatively few number of people that fulfil the YFAS criterion [16, 17, 21•].

## Eating Behaviour in Self-Perceived Food Addicts

While the majority of self-perceived food addicts do not fulfil an existing clinical criterion for food addiction (i.e. the YFAS), there is emerging evidence to suggest that these individuals may have increased aberrant patterns of eating. For example, one study revealed that self-perceived food addicts scored higher on various measures of pathological eating compared to self-perceived nonaddicts [16], and we found an increased prevalence of self-perceived food addiction in those with increased BMI [18••]. Furthermore, several experimental studies have revealed increased desire for chocolate and greater ad libitum chocolate consumption in self-perceived chocolate addicts relative to chocolate nonaddicts [24–26].

Building upon these findings, we recently explored food reward and intake in self-perceived food addicts and nonaddicts [21•]. Food reward was assessed using an operant response task and by asking participants how much they would be ‘willing to pay’ for and the strength of their current ‘desire to eat’, a portion of food. These measures were completed for two high-fat foods and two low-fat foods when participants were hungry and again when they were satiated. Participants were then given ad libitum access to a high-fat and a low-fat food. Despite demonstrating no elevated levels of hunger or ‘liking’ for the test foods, self-perceived food addicts showed increased food reward (as indicated by ‘desire to eat’ ratings) for both the high- and low- fat foods, when they were hungry and satiated, compared to self-perceived nonaddicts. Further, self-perceived food addicts consumed more calories during the taste task. In particular, and consistent with beliefs about the addictiveness of certain foods (e.g. 14), self-perceived food addicts consumed more calories from the high-fat food, but not the low-fat food. These findings suggest that self-perceived food addicts have problematic patterns of eating that may go undetected by an existing measure of addictive eating (i.e. the YFAS). Notably, increased calorie intake in self-perceived food addicts was explained by increased dietary disinhibition and diminished dietary restraint (as indicated by a variety of eating-trait questionnaires). This is consistent with dual-process theories of overeating and addiction which suggest that the overconsumption of food and drugs is driven by increased appetitive motivation *and* a diminished ability for self-control [27, 28].

## Food Addiction as a Self-Serving Attribution

Psychosocial theories consider the role of psychological and societal influences in the formation of food addiction attitudes and beliefs. From this perspective, it has been suggested that, rather than reflecting addictive patterns of eating per se, self-perceived food addiction may reflect attempts to minimise perceptions of blame and guilt associated with overeating. Specifically, Rogers and Smit [29] propose that labelling oneself a ‘food addict’ may arise following failed attempts to restrict one’s intake of highly palatable but unhealthy (‘naughty but nice’) foods. By insinuating that such lapses in self-control are the result of a physiological inevitability, Rogers and Smit [29] suggest that the concept of food addiction may help to alleviate feelings of guilt and personal responsibility. This theory is consistent with the core principles of ‘attribution theory’ which posits the tendency for individuals to make attributions for negative outcomes which minimise the role of internal and/or controllable factors (e.g. personal choice) and maximise the role of external and/or uncontrollable factors (e.g. environmental or biological factors). Based on this theory, Davies [30] proposes that the concept of ‘addiction’ minimises perceptions of blame by portraying the drug user or overeater as a ‘helpless victim of disease’. Indeed, there is evidence to suggest that addiction-based explanations may be effective in reducing obesity-related blame [31], although this has not been consistently demonstrated [32].

In a recent study [33], we tested this theory by examining whether those who were led to feel guilty following eating would be particularly likely to identify as food addicts and to attribute their eating to the foods’ addictive properties. Feelings of guilt following eating were manipulated by leading participants to believe that they had consumed more than (high-guilt condition), less than (low-guilt condition), or roughly the same (control condition) amount of high-calorie foods than previous bogus participants and relative to their own estimated intake. Participants were then asked to indicate the extent to which they perceived themselves to be ‘food addicts’ and to rank ten ‘reasons for eating’ from most to least influential. In particular, we were interested in the rank assigned to a ‘foods were addictive’ attribution. It was predicted that participants in the high-guilt condition would be more likely to label themselves ‘food addicts’ and would assign a lower rank (indicative of being more influential) to the ‘foods were addictive’ attribution, compared to those in low-guilt and control conditions. Contrary to expectation, there was no effect of condition (i.e. high-guilt vs. low-guilt vs. control) on food addiction attributions. However, across the whole sample of participants, those with higher levels of eating-related guilt assigned a lower rank (indicative of being more influential) to the ‘foods were addictive’ reason for eating. Importantly, the rank assigned to this attribution was unrelated to actual or perceived calorie intake. Thus, while these findings do not fully support the concept of food addiction as an ‘attribution’, they do suggest that beliefs about the addictive potential of foods may be more closely related to feelings of guilt than to actual calorie intake. More research is needed to directly test this hypothesis.

## The Consequences of Food Addiction Beliefs

Psychosocial perspectives on food addiction also consider the *consequences* of food addiction beliefs on eating behaviour [9•]. This is of potential concern as biological explanations for obesity may lead people to believe that their weight is uncontrollable [34] and consequently undermine their efforts to lose weight. Indeed, believing that one is in control of one’s behaviour (i.e. self-control beliefs) has been found to be important in the initiation of health behaviours [35], and those with increased self-control beliefs are less likely to snack on sugary foods [36] and are more likely to act upon healthy eating intentions [37] and lose weight [38].

Research has also demonstrated a deleterious effect of biological explanations for obesity on dietary behaviour. Across a series of studies, Hoyt et al. [39] found that participants who read an article which explained that ‘obesity is a disease’, made higher calorie food choices compared to those who read a control article or one which stated that ‘obesity is not a disease’. In particular, this effect was observed in overweight and obese participants, but *not* in healthy weight participants, and was mediated by the effect of the disease message on decreased levels of body dissatisfaction. A similar finding was observed by Dar-Nimrod et al. [40] who found that participants who read a genetic explanation for obesity perceived weight to be less controllable and consumed more calories in a subsequent taste task, compared to those who read a psychosocial explanation for obesity or a control article.

In contrast, however, a recent study uncovered no effect of a ‘food addiction is real’ message, relative to a ‘food addiction is a myth’ message, on subsequent calorie intake [17]. This may be due to that fact that, unlike disease- or genetic-based explanations for obesity, food addiction beliefs do not necessarily imply a lack of control over weight. Indeed, Lee et al. [41] found that, despite strong endorsement of the food addiction concept (by 86% of the sample), 55% of those surveyed believed that overeating and weight gain are within personal control. Furthermore, following a survey of 570 American adults, DePierre et al. [42••] found that food addiction was perceived to be more of a behavioural choice compared to other addictions, such as alcoholism.

Taken together, these findings suggest that food addiction messages may not elicit the same negative effects on self-control beliefs and eating behaviour as more general biological explanations of obesity. On the contrary, there is initial evidence to suggest that perceiving oneself to be a food addict may actually be *helpful* for those attempting to reduce their overeating tendencies. For example, members of the self-help group, Overeaters Anonymous, are encouraged to view their overeating as an ‘addiction’ and to avoid exposing themselves to their ‘problem’ foods. Qualitative reports suggest that this perspective helps to alleviate members’ feelings of eating-related guilt and shame and promotes a sense of personal responsibility for members’ own recovery [43, 44].

Our recent empirical study also suggests that believing oneself to be a food addict may have initial helpful effects on eating behaviour [45••]. Over two studies, participants were led to believe that they had scored either high, low, or average on an ostensible measure of food addiction. Following this, a measure of dietary concern was taken, and participants were given ad libitum access to high-fat foods during a taste task. The amount of time participants spent tasting and rating the foods was covertly recorded. Results revealed that participants who were led to believe they had scored high on food addiction consumed fewer calories during the taste task compared to those who were in low and average conditions. Further analyses revealed that this was due to increased levels of dietary concern in the high-food addiction condition following the food addiction feedback and a subsequent reduction in the amount of time these participants spent tasting the foods. Although conducted in a laboratory setting, these findings have potential real-world implications. For example, it is possible that perceiving oneself to be a food addict may encourage individuals to avoid exposing themselves to tempting situations, such as the supermarket confectionary aisle or the buffet table at a party.

However, before drawing definitive conclusions regarding the consequences of food addiction beliefs, it is necessary to consider their longer-term impact. More specifically, while food addiction beliefs may encourage individuals to restrict their food intake in the short term, previous research suggests that attempts to restrict food intake over longer time periods can exacerbate cravings and promote disinhibited eating [46, 47]. Indeed, our previous findings suggest that self-perceived food addiction is associated with *increased*, rather than *decreased*, levels of dietary disinhibition and BMI [18••, 21•].

**Fig. 1:**
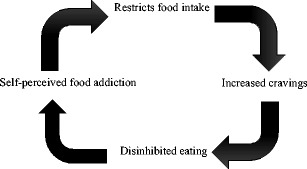
A proposed self-perpetuating relationship between self-perceived food addiction and food intake. Belief that one is a food addict leads to an initial restriction of high-calorie or unhealthy foods. This restriction increases cravings and eventually leads to overconsumption of restricted foods. This reinforces the individual’s food addiction beliefs

In order to reconcile these disparate findings, we propose a self-perpetuating relationship between food addiction beliefs and food intake (Fig. 1). Specifically, it is proposed that, over the short term, perceiving oneself to be a food addict elicits attempts to avoid particular ‘problem’ foods. In turn, and consistent with previous evidence [46, 47], it is thought that attempts at dietary restriction will strengthen cravings and eventually lead to the over consumption of the forbidden foods. Finally, these increased cravings and failed attempts at dietary restriction may reinforce perceptions of oneself as a ‘food addict’, thus resulting in further attempts at dietary restriction. Future research is required to explore this potential self-perpetuating relationship between food addiction beliefs and high-calorie food consumption. In particular, it would be informative to extend our previous findings [45••] by examining the longer-term effects of manipulating food addiction beliefs on intentions to restrict food intake and the effects of this on food cravings and subsequent consumption. Furthermore, research should examine the possibility that labelling oneself a food addict may help to alleviate feelings of guilt following regular and repeated episodes of overeating.

## Conclusion

The concept of food addiction is widely endorsed throughout the lay public, and many people attribute their food cravings and overeating tendencies to an ‘addiction’ to foods that are high in fat, salt, and sugar. While the majority of these individuals do not meet an existing substance-based definition of food addiction, they nonetheless demonstrate increased food reward and calorie intake within the lab. Further, these behaviours appear to be driven by increased appetitive motivation and diminished self-control around food, consistent with dual-process models of overeating and addiction. As such, self-perceived food addicts appear to represent a population that is at particular risk of overeating and weight gain and may therefore benefit from early dietary interventions which aim to increase food-related self-control and minimise temptation for high-calorie foods.

It is necessary for future research to reconcile findings of aberrant eating behaviour in self-perceived food addicts, with those which suggest that food addiction beliefs may have short-term *helpful* consequences for eating behaviour. In particular, given the popularity of the food addiction concept throughout the lay community and the promotion of food addiction messages within weight management groups, examining the longer-term consequences of food addiction beliefs is an especially important avenue for future research.
